# Effects of Deformation Parameters on Microstructural Evolution of 2219 Aluminum Alloy during Intermediate Thermo-Mechanical Treatment Process

**DOI:** 10.3390/ma11091496

**Published:** 2018-08-22

**Authors:** Lei Liu, Yunxin Wu, Hai Gong

**Affiliations:** 1 Light Alloy Research Institute, Central South University, Changsha 410083, China; Larill@csu.edu.cn; 2 State Key Laboratory of High Performance Complex Manufacturing, Central South University, Changsha 410083, China; csu2019@163.com

**Keywords:** 2219 aluminum alloy, intermediate thermo-mechanical treatment, storage energy, CuAl_2_ phase, grain refinement

## Abstract

To explore the effective way of grain refinement for 2219 aluminum alloy, the approach of ‘thermal compression tests + solid solution treatment experiments’ was applied to simulate the process of intermediate thermo-mechanical treatment. The effects of deformation parameters (i.e., temperature, strain, and strain rate) on microstructural evolution were also studied. The results show that the main softening mechanism of 2219 aluminum alloy during warm deformation process is dynamic recovery, during which the distribution of CuAl_2_ phase changes and the substructure content increases. Moreover, the storage energy is found to be decreased with the increase in temperature and/or the decrease in strain rate. In addition, complete static recrystallization occurs and substructures almost disappear during the solid solution treatment process. The average grain size obtained decreases with the decrease in deforming temperature, the increase in strain rate, and/or the increase in strain. The grain refinement mechanism is related to the amount of storage energy and the distribution of precipitated particles in the whole process of intermediate thermal-mechanical treatment. The previously existing dispersed fine precipitates are all redissolved into the matrix, however, the remaining precipitates exist mainly by the form of polymerization.

## 1. Introduction

2219 aluminum alloy, which has the advantages of high strength and resistance to stress corrosion, good weldability, and service performance, was considered as the third generation of space materials and has been widely used in aerospace industry [1]. Its good mechanical properties are closely related to microstructural characteristics and depend largely on their chemical composition, processing history, and heat treatment process [2]. During the manufacturing process, strength and plasticity targets are two of the most important performance parameters to be achieved. For 2219 aluminum alloy that can be strengthened by heat treatment, solid solution strengthening and precipitation strengthening are effective means to improve its strength [3,4,5]. Concerning the improvement of ductility, the grain refinement approach may be often applied [6]. Also, grain refinement is the only method that can effectively improve both the ductility and the strength of the materials [7,8]. So, factories attach great importance to controlling the grain size, especially for those metals and alloys without phase change [9]. Therefore, the theory and technology of grain refinement for 2219 aluminum alloy are of great significance to be investigated.

Thermo-mechanical treatment (TMT), an advanced and coupled metallurgical technology, has an unparalleled advantage in refining grain and improving plasticity and the comprehensive properties of metals and alloys [10,11]. It is mainly through coupling the dislocations and other defects produced by deformation with the morphology and distribution of the precipitated phase during heat treatment that the grain refinement is achieved, thus improving the strength and ductility of the material [12,13]. According to the study of E.D. Russo [14,15], TMT is defined as the intermediate thermo-mechanical treatment (ITMT) when the precipitated phase only acts as auxiliary particles, such as the core of the recrystallized nucleation or the obstructor of grain boundary migration (GBM) during the recrystallization process. Many scholars [16,17,18,19,20,21,22,23,24,25,26,27,28,29,30,31,32,33,34,35,36,37] have made extensive efforts on the process of ITMT. Some scholars [16,17] focused on the traditional combination of cold deformation (CD) and recrystallization annealing treatment, also known as thermal mechanical processing (TMP). J. Waldman [18] proposed a new FA-ITMT process with ‘subsection cooling and thermal insulation homogenization treatment + CD + solid solution treatment (SST)’. B.R. Ward [19] improved the process to enlarge its application. J. Wert [20] developed a new superplastic pretreatment (SPPT) process and the grain size of the 7050-aluminum alloy was refined to 10 μm. Although the technological measures adopted before ’CD + SST’ are different, all the purposes are to precipitate dispersed second-phase particles. Some studies [21,22] focused on the effect of precipitation on the recrystallization process (i.e., recrystallization kinetics, texture evolution, grain size, etc.). With regard to the effect of ITMT parameters on the microstructure evolution, S. Primig [23] found that the grain size of the recrystallization solution treatment (RST) process increases with the increasing of heating rate, J. Waldman [10] found that the grain refinement can be achieved by increasing the RST temperature, and H. Yoshida [24] found that RST period has little effect on the grain refinement. The deformation parameters have a great influence on the grain size and orientation and the distribution of the precipitated phase, so it is an important part of the ITMT process. However, the study on the effects of deformation parameters were rarely reported. In addition, most of the studies on ITMT processes are focused on the optimization and improvement of 7000 serials aluminum alloy [25,26,27,28], Al-Li alloy [29], and 6000 series aluminum alloys [30,31], Ni-rich Ti-51.5 at.% Ni shape memory alloy [32], Ti-28Nb-35.4Zr alloy [33], direct-quenched low-carbon strip steel [34], commercial austenitic stainless steel [35,36,37], and so on. However, the grain refinement effect of the ITMT process for 2219 aluminum alloy is rarely reported. Therefore, it is urgent to study the effects of deformation parameters on microstructural evolution of 2219 aluminum alloy during the ITMT process.

In this paper, the ITMT process of 2219 aluminum alloy was simulated by warm compression + solution treatment experiment. The evolution of grain size and the second phase on 2219 aluminum alloy during warm deformation and solution treatment were studied. In addition, the grain refinement mechanism of 2219 aluminum alloy and the influence of the deformation parameters were discussed in detail. Finally, the optimal deformation parameters of ITMT were summarized.

## 2. Materials and Methods 

**Figure 1:**
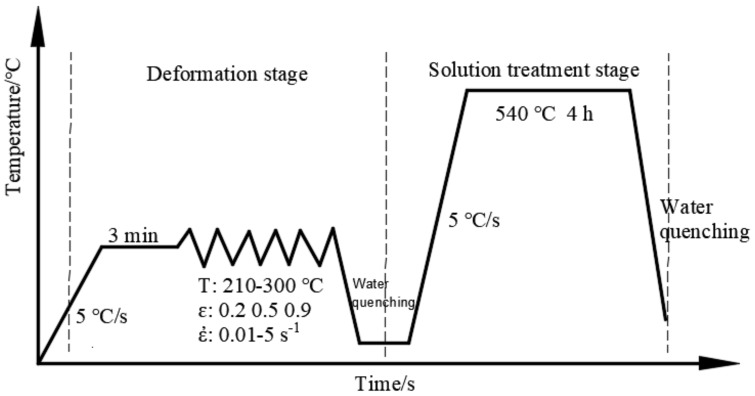
Experimental scheme of intermediate thermo-mechanical treatment process.

To explore the influence of deformation parameters (temperature: T, strain: $\varepsilon ,\;\mathrm{strain}\;\mathrm{rate}:\;\dot{\varepsilon }$) on the microstructural evolution during the process of ITMT (warm deformation (WD) + SST), the experimental scheme was adopted as shown in Figure 1.

The warm deformation stage was conducted on the Gleeble-3500 thermal simulator unit (Dynamic Systems Inc., New York, NY, USA). Different deformation parameters were arranged with the temperature range of 210–300 °C, the strain rate range of 0.01–5 s^−1^, and the strain range of 0–0.9. The samples were water quenched immediately after the deformation stage to retain the deformed microstructure. Subsequently, the samples were heated to 540 °C in a quenching furnace and kept for 4 h during the solution treatment stage, and the water quenched was also adopted to preserve the solution-treated microstructure.

The samples were taken from 2219 aluminum alloy plate after hot rolling, and their chemical composition is shown in Table 1. All the samples were machined to cylinder shape with the height of 15 mm and the diameter of 10 mm. During the warm compression stage, some lubricants were added to the ends of the samples to reduce friction with the dies. After the experiment, the samples were cut along the deformation direction for microstructural observation by scanning electron microscope (SEM, Oxford Instruments Inc., Oxford, UK) and electron back-scattered diffraction (EBSD, Helios Nanolab 600i, FEI Company, Hillsboro, OS, USA) method. The different phases existing in present samples under different TMT stages were detected by an X-Ray diffractometer (XRD, Advance D8, Bruker Beijing Scientific Technology Co., Ltd., Beijing, China). The samples for SEM were ground and mechanically polished, and the samples for EBSD were ground and mechanically and electrolytically polished. The electrolyte used was the mixture of nitric acid and methanol solution with volume ratio of 3:7. The samples were electrolyzed under the voltage of 22 V and kept for 55 s. The results were processed by Channel 5 software (HKL Technology, Inc., Danbury, CT, USA), in which the high angle grain boundaries (HAGB: misorientation >15°) were expressed in coarse black solid lines, and the low angle grain boundaries (LAGB: misorientation >2°) were expressed in fine white solid lines.

## 3. Results and Discussion

### 3.1. Initial Micrographs Analysis of the Undeformed Sample

**Figure 2:**
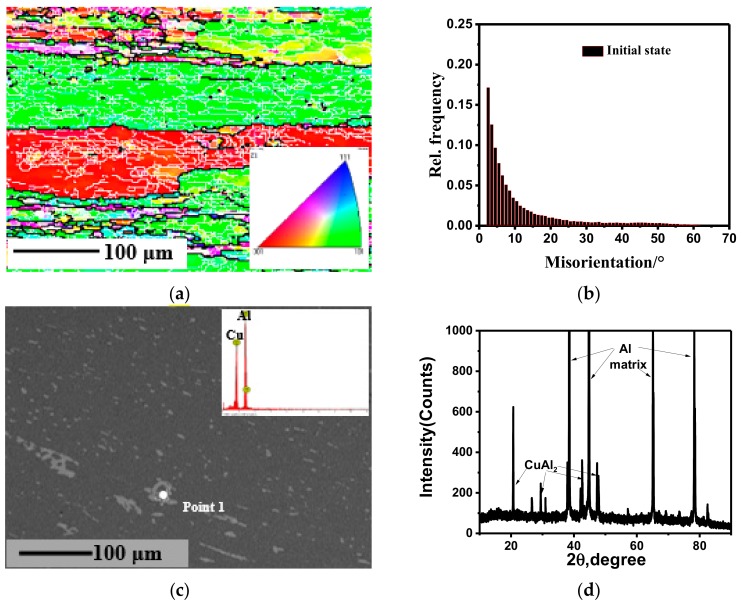
Initial microstructure of 2219 aluminum alloy used in the experiment: (**a**) EBSD micrograph; (**b**) the frequency distribution of misorientation; (**c**) SEM micrograph and EDS analysis of Point 1; (**d**) XRD analysis.

The microstructures of the samples in the initial state are shown in Figure 2. As shown in Figure 2a, the initial grain of the samples presents a typical elongated state after hot rolling, with the length >500 μm and the width >100 μm, which may make the material exhibit anisotropic properties. Some fine recrystallized grains are found at local grain boundaries, which indicates dynamic recrystallization initiated during hot rolling. A large number of substructures (i.e., fine white solid lines) can be found inside the grain. This is because many dislocations accumulate and intertwine in local areas as a result of the dislocation density increasing and their interaction effects during the previous large plastic deformation, thus forming an uneven distribution and making a grain into many small crystal blocks (i.e., subgrains) with slightly different misorientations. Moreover, the inverse pole figure (IPF) shows that (001) and (101) grains are dominant and (111) grains are few. By the misorientation analysis (Figure 2b), LAGB content (77.6%) dominates, far exceeding that of HAGB (22.4%). In addition, as shown in Figure 2c, the distribution of the second phase in the solid solution matrix is approximately dispersed, and its particles are approximately spherical with the size of about 10 μm. However, a few parts of the precipitated phase are connected to lumps or chains. The second phase can be determined to be CuAl_2_ (θ) phase by energy disperse spectroscopy (EDS, Oxford Instruments Inc., Oxford, UK) and XRD analysis.

### 3.2. Effects of Deformation Parameters on the Deformed Microstructure during WD Process

#### 3.2.1. Effects on Flow Behavior

**Figure 3:**
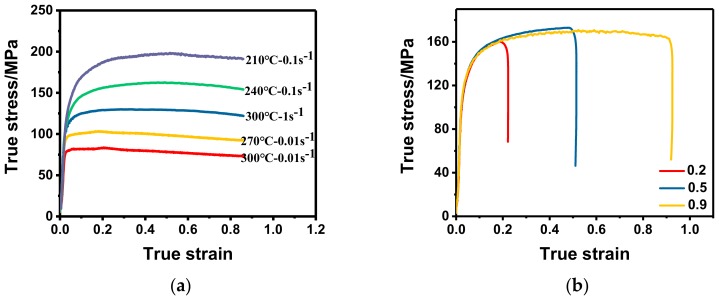
True stress–strain curves under: (**a**) different temperatures and strain rates; (**b**) the strain of 0.2, 0.5, and 0.9.

The true stress–strain curves of 2219 aluminum alloy under different warm deformation conditions are shown in Figure 3. It can be clearly seen that the flow stress is very sensitive to the deformation parameters and increases with the decrease in temperature and/or the increase in strain rate. On one hand, the slip system is limited and the dislocation accumulation and entanglement are serious at lower temperature, which makes the flow stress higher. With the increase of temperature, the diffusion of the vacancy and the slip and climbing of dislocation become easier, thus the dynamic softening effect is enhanced and the flow stress is reduced [38]. On the other hand, the lower strain rate provides sufficient time for dislocation movement, making the dynamic recovery (DRV) more thorough, and the softening effect becomes more obvious [39,40].

In addition, each true stress–strain curve can be divided into two stages: Short work hardening stage and long stable flowing stage. This characteristic marks obvious dynamic recovery process. In the stage of work hardening, the flow stress increases rapidly with the increase of strain. It is due to the rapid increment and accumulation of dislocation, which makes the resistance of the deformation increase sharply, and the dynamic softening effect is too weak to make up for the work hardening effect [41]. In the stage of stable flowing, the flow stress is almost invariable, which is due to the counteraction of the work hardening effect and dynamic softening effect in the deformation process [42]. That is to say, the proliferation and reduction of dislocations have reached a kind of equilibrium. In particular, the flow stresses of the 2219 aluminum alloy decrease slightly under the conditions of 270 °C and 300 °C-0.01 s^−1^, which may characterize the occurrence of partial dynamic recrystallization.

According to the analysis above, the evolution of increment and disappearance of dislocation determine the magnitude of flow stress, and the mathematical relationship between them can be expressed as follows [43]:

(1) $$\sigma =\alpha \mu b\sqrt{\rho }$$

where *α* is the material constant, *μ* is the shear modulus, *b* is the Burgers vector, *ρ* is the dislocation density, and *σ* is the flow stress (MPa). Therefore, the flow stress is proportional to the root of dislocation density.

In addition, the relationship between storage energy and dislocation density can be expressed as follows:

(2) $$E_{st}=c\mu \rho b^{2}$$

where *E_st_* is the storage energy (kJ) and *c* is the material parameter. It can be seen that the storage energy is proportional to the dislocation density. According to Equations (1) and (2), the relationship between storage energy and flow stress can be obtained and shown as follows:

(3) $$E_{st}=c_{1}\mu^{-1}\sigma^{2}$$

where *c*_1_ = *c*/*α*^2^. It can be seen from Equation (3) that the storage energy is proportional to the square of the flow stress. That is to say, the storage energy also decreases with the increase in temperature and the decrease in strain rate.

#### 3.2.2. Effects on Subgrain Evolution

**Figure 4:**
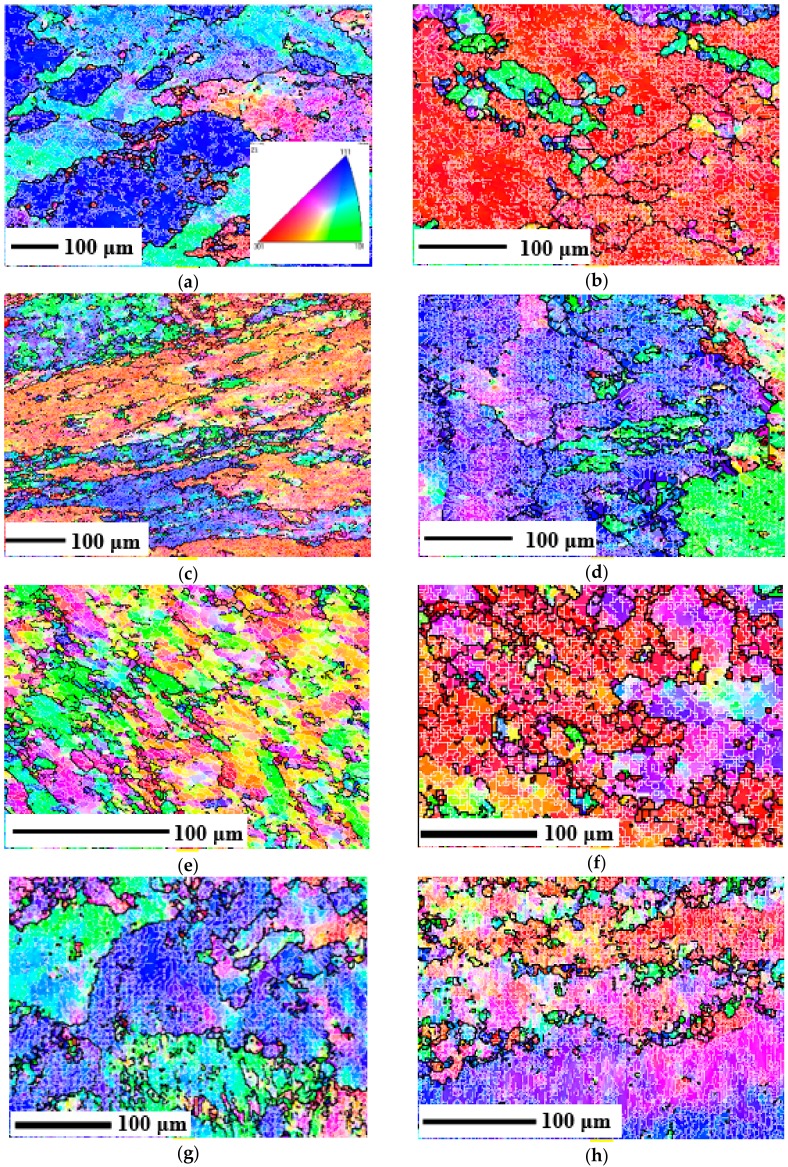
EBSD micrographs of the deformed samples under the condition of: (**a**) 240 °C-0.3 s^−1^-0.2; (**b**) 240 °C-0.3 s^−1^-0.5; (**c**) 240 °C-0.3 s^−1^-0.9; (**d**) 210 °C-0.01 s^−1^-0.9; (**e**) 270 °C-0.01 s^−1^-0.9; (**f**) 300 °C-0.01 s^−1^-0.9; (**g**) 300 °C-0.1 s^−1^-0.9; (**h**) 300 °C-1 s^−1^-0.9.

**Figure 5:**
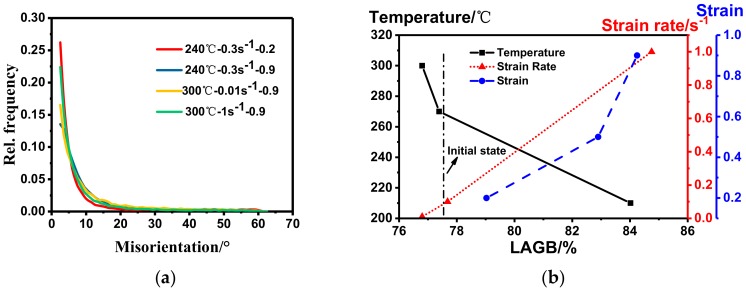
(**a**) Frequency distribution of misorientation under different deforming conditions; (**b**) the relationship between the content of LAGBs and deforming temperature, strain rate, and strain.

The EBSD micrographs of 2219 aluminum alloy after different deformation conditions are shown in Figure 4. It is observed that the microstructures obtained show obvious characteristics of DRV, which is in accordance with the true stress–strain curves of Figure 3. The cutting surface is a random diameter plane when the deformed sample is cut by wire electrode discharge machining (WEDM). Therefore, the grains exhibit various forms as a result of the different planes selected. But all the grains have different degrees of fragmentation and deformation because all of them are compressed. According to the IPF, it can also be seen that the grains have different dominant orientations in different cutting planes. In addition, many LAGBs are observed inside the grains, which shows that sufficient storage energy is introduced and the driving force for recrystallization can be provided with thermal energy in the subsequent annealing process [44]. According to the probability density function of the dislocations (Figure 5a) and the volume ratio of LAGB and HAGB (Table 2), it can be seen that the distribution of the misorientation remains almost the same and LAGBs occupy the dominant content. However, the quantitative content of LAGBs is changed, and it is closely related to the deformation parameters.

From Figure 4a–c, it can be seen that the area of HAGBs increases with the increase of strain due to grain deformation. In addition, the content ratio of LAGB and HAGB increases from 79.0% to 84.3%. This shows that the density of dislocation cell and the content of LAGB increase with the increase of strain, so more energy can be stored in the dislocations on the subgrain boundaries. Therefore, the storage energy gradually increases with the increase in strain [45].

From Figure 4d–f, it can be seen that with the increase of the deformation temperature, not only the structures of initial grains are changed, but also some new fine recrystallized grains (Figure 4e) and the grown equiaxed recrystallization grains (Figure 4f) are formed near the grain boundaries, which is due to the continuous dynamic recrystallization (CDRX) occurring by the continuous lattice rotation near the grain boundaries [46,47]. This is consistent with the decreasing trend of the corresponding flow curves at large strains in Figure 3. Meanwhile, according to Figure 5b and Table 2, the content ratio of the LAGBs against HAGBs under the deformed conditions of 270 °C-0.01 s^−1^ (77.4%) and 300 °C-0.01 s^−1^ (76.8%) are lower than the initial state (77.6%). It is proved again that DRX of 2219 aluminum alloy has occurred at this condition. It is also observed that only a small portion of the recrystallized grains are found in local areas and the initial deformed grains occupy the dominant contents, which indicates the main dynamic softening mechanism is DRV. Moreover, the driving force for DRV increases with the increase in temperature and dislocation density [48]. Therefore, DRV proceeds fiercely and the dislocation density becomes lower as the temperature increases, so storage energy will be reduced.

From Figure 4f–h and Figure 5b, and Table 2, it is observed that the content ratio of LAGBs against HAGBs increases from 76.8% to 84.8% with the increase in strain rate at the deformed temperature of 300 °C, which shows the increasing contents of substructures. This is because the increase in strain rate makes the deformation time shortened and DRV process incomplete. Therefore, the dislocation density increases greatly and the storage energy can be increased with the increase in strain rate.

#### 3.2.3. Effects on the Distribution of Precipitated Phase

**Figure 6:**
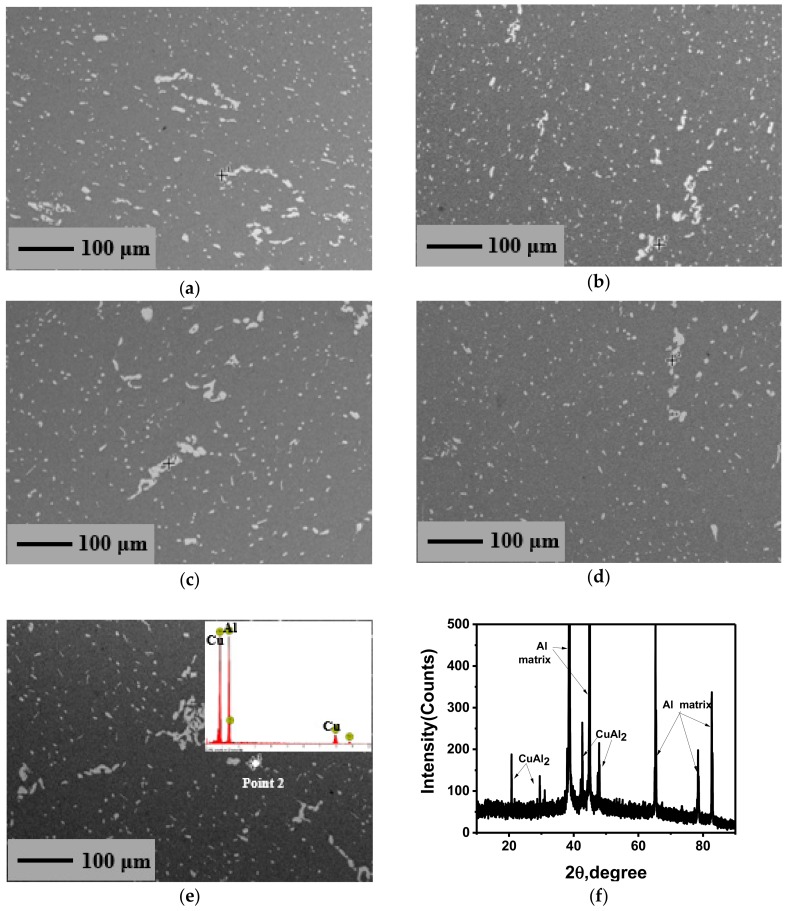
SEM micrograph of the deformed samples under the condition of: (**a**) 240 °C-0.3 s^−1^-0.2; (**b**) 240 °C-0.3 s^−1^-0.9; (**c**) 210 °C-0.01 s^−1^-0.9; (**d**) 300 °C-0.01 s^−1^-0.9; (**e**) 300 °C-0.1 s^−1^-0.9 and EDS analysis of Point 2; (**f**) XRD analysis of the deformed samples.

The SEM micrographs of the deformed samples are shown in Figure 6. It can be seen that the distribution of the second phase is relatively dispersed and small parts of the second phase are united together, which is similar to the initial state (Figure 1c). This indicates that the second phase of 2219 aluminum alloy cannot be broken apart during warm deformation. By EDS and XRD analysis in Figure 6e,f, the precipitated phase is still CuAl_2_ (θ) phase. It can be observed that deformation can affect the shape and size of the precipitated phase [49,50], but the content of the precipitated phase is only determined by the solubility under different temperatures in terms of thermodynamics.

The precipitates in 2219 aluminum alloy play an important role in the process of ITMT, and the relationship between precipitates and substructures may be the main mechanism of grain refinement [51]. Studies [52,53] show that a large number of dislocations and local deformable zones are formed around the large precipitated particles, and the deformed energy is introduced. Thus, the recrystallized core will be formed during the subsequent SST process (i.e., particles stimulate nucleation, PSN). The dispersed small particles apply resistance to the migration of grain boundaries or subgrain boundaries to limit the growth of recrystallized grains during the SST process [52], and finally refined grains will be obtained [53].

As shown in Figure 6a,b, the condensation of the precipitated phase is slightly relieved and the distribution is more diffuse as the strain increases. This is due to the crush of large lump precipitates and the movement of particles as the strain increases. In addition, the formation and spheroidization of the second phase can be promoted by the introduction of crystal defects (vacancies and dislocation, etc.) during warm deformation. This can effectively impede the movement of dislocations and promote the formation of dislocation walls, and eventually form a large number of polygonal substructures through DRV [54]. From Figure 6c,d, it can be seen that the content of θ phase is slightly reduced, the condensation phenomenon is slightly relieved, and the distribution is more diffuse as the temperature increases. This is because the solubility of CuAl_2_ increases with the increase in temperature, which makes a part of the precipitates resolve to the matrix and thus reduces the content of the dispersed phase. In addition, solute atoms diffuse and migrate more readily and the alloy has a better plastic mobility as the temperature increases, thus making the distribution more uniform. From Figure 6d,e, it is known that large precipitate lumps become serious and the distribution is relatively nonuniform as the strain rate increases. This is due to the shorter deformation time and poor fluidity at the high strain rate.

### 3.3. Effects of Deformation Parameters on the Microstructures during SST Process

#### 3.3.1. Effects on Grain Refinement

**Figure 7:**
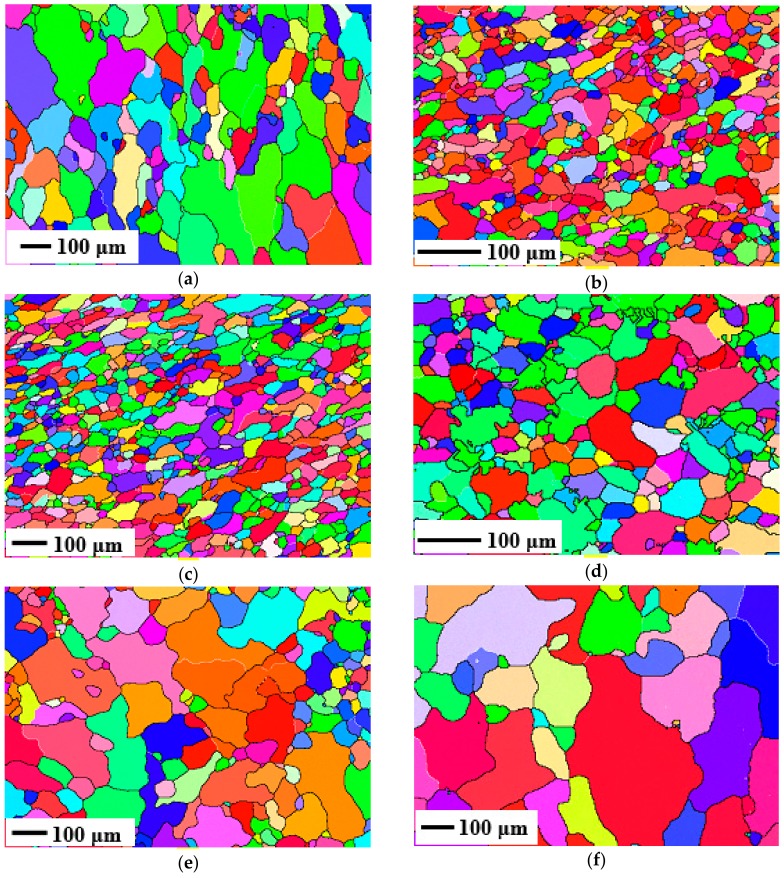

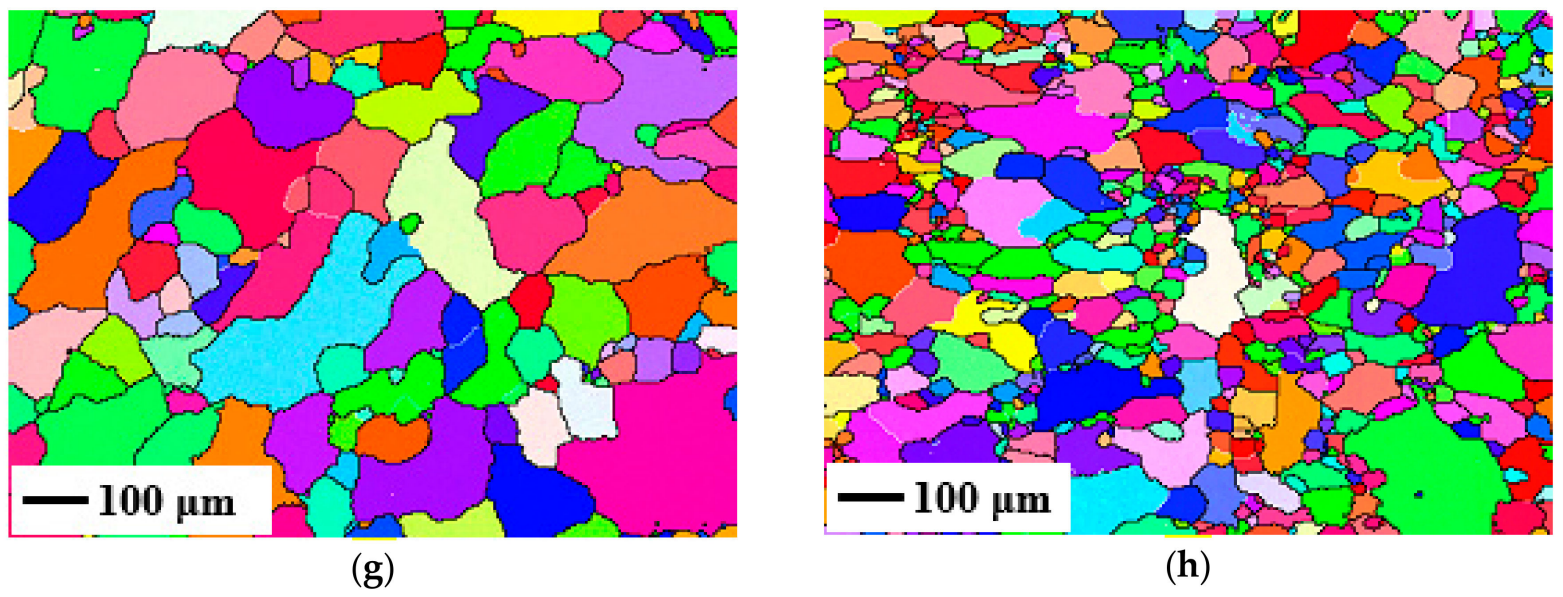
EBSD micrograph of the solution-treated samples under the condition of: (**a**) 240 °C-0.3 s^−1^-0.2; (**b**) 240 °C-0.3 s^−1^-0.5; (**c**) 240 °C-0.3 s^−1^-0.9; (**d**) 210 °C-0.01 s^−1^-0.9; (**e**) 270 °C-0.01 s^−1^-0.9; (**f**) 300 °C-0.01 s^−1^-0.9; (**g**) 300 °C-0.1 s^−1^-0.9; (**h**) 300 °C-1 s^−1^-0.9.

**Figure 8:**
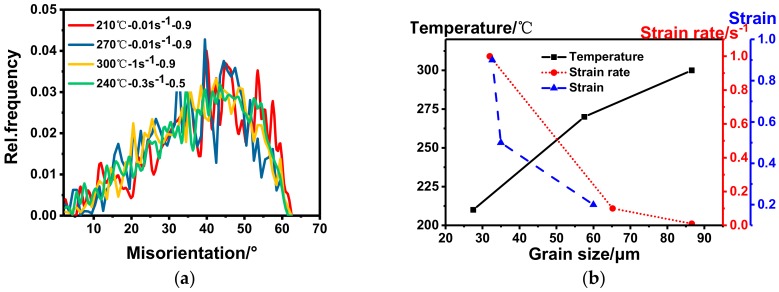
(**a**) Frequency distribution of misorientation and grain size under different conditions; (**b**) the relationship between grain size and temperature, strain rate and strain, respectively.

The EBSD microstructures of the samples after SST are shown in Figure 7. It can be clearly seen that (001), (101), and (111) grains are randomly arranged, and there are no deformed microstructures, which indicates that complete static recrystallization (SRX) has occurred. The elongated (or squashed) and broken grains are reproduced and grown to new uniform and fine equiaxed grains because of the increase of the atomic diffusion capacity when the deformed metal is solution treated at a high temperature. In addition, all the substructures have almost disappeared and the frequency distribution of the misorientations is shown in Figure 8a. It can be seen that the distribution curves of misorientations are similar to each other and the content of LAGBs is less than 8%. However, the grain size under different deformation parameters is quite different. The expectation (EX) and the deviation coefficient (EX/s, s: Standard deviation) of the grain size by mathematical statistics are shown in Table 3. It can be seen that all the samples are refined to different degrees compared with the initial state, which indicates that the deformation parameters have a significant effect on grain refinement during the recrystallization process. Moreover, the grain size during the SRX process can be determined by the following formula:

(4) $$d=2R=2\int_{0}^{t_{R}}vdt=2\sqrt[{4}]{\frac{3v}{\pi N}}$$

where *d* is the diameter of grains, *N* is the nucleation rate, and *v* is the growth rate. It can be seen that the grain size decreases with the increase in nucleation rate and/or the decrease in growth rate.

From Figure 7a–c and Table 3, the grain size is refined from 60 μm to 33 μm after SST, and the size deviation coefficient is becoming smaller as the amount of deformation increases. This is due to the fact that when the amount of deformation is small, only a few grains are deformed and the deformation distribution inside the metal is quite uneven, so the amount of grain nucleation is relatively less and the new grains can grow up quickly as a result of the different deviations in grain sizes. Thus, the coarse recrystallized grains are obtained. As the amount of deformation increases, more crystal defects of the alloy and more unstable substructures will be introduced, thus more storage energy will be obtained, which motivates the development of SRX and the increase of the nucleation rate during the SST process. Moreover, the refined and uniformly distributed precipitates can effectively hinder the atoms’ diffusion and grain boundary migration, which can limit the growth of recrystallized grains. Therefore, the grain size decreases with the increase in strain. It is also found that the grain size under the strain of 0.5 and 0.9 is little changed. The main reason is that the smaller precipitation phases cannot become the core of the recrystallization, and the nucleation density cannot be increased with the continuous increase in the strain, so the grains cannot be further refined [55].

As shown in Figure 7d–f and Table 3, the average grain size increases gradually with the range of 27 μm to 87 μm, and the deviation coefficient increases from 0.58 to 0.92 with the increase in deforming temperature. This is related to the amount of storage energy. According to the previous discussion, the storage energy decreases with the increase in deforming temperature, so the nucleation rate of SRX decreases with the increase in deforming temperature during the SST process. Moreover, the amount of the precipitated phase decreases with the increase in deforming temperature, which will lead to the decrease in the nucleation rate and weaken the pinning effect. Therefore, the average grain size will increase with the increase in deforming temperature.

As shown in Figure 7f–h and Table 3, the recrystallized grain size decreases gradually from 87 μm to 32 μm with the increase in strain rate and the deviation coefficient is also reduced from 0.92 to 0.73. This is also related to the effect of storage energy and the precipitated phase. According to the previous discussion, the storage energy increases with the increase in strain rate, so the nucleation rate of SRX also increases with the increase in strain rate. In addition, although the nucleation rate is high at high strain rate, the distribution of the precipitated phase is even worse with the increase in the strain rate, which results in the inhomogeneity of the growth and the final uneven distribution of grain size.

Generally, the relationship between yield stress and average grain size of alloys can be characterized by the Hall–Petch formula [8,56]:

(5) $$\sigma_{s}=\sigma_{0}+k_{y}d^{-1/2}$$

where *σ_s_* is the yield stress of the material, *σ*_0_ and *k_y_* are material constants, and *d* is the average grain size. It can be seen that yield stress increases with the decrease in grain size.

According to the study of Wert [57], the coefficient *k_y_* in the formula is about 0.12, which means that the effect of recrystallized grain size on yield stress of 2219 aluminum alloy is very small. Thus, the yield stress change caused by grain refinement from 87 μm to 27 μm can be calculated as ∆*σ_s_* = *k_y_* × ∆*d* ^(−1/2)^ = 0.01 MPa, which indicates that the strengthening effect caused by grain refinement is negligible, and the precipitation strengthening is the main strengthening mechanism of 2219 aluminum alloy. This is consistent with the existing conclusions [58,59].

#### 3.3.2. Effects on Precipitated Phase

**Figure 9:**
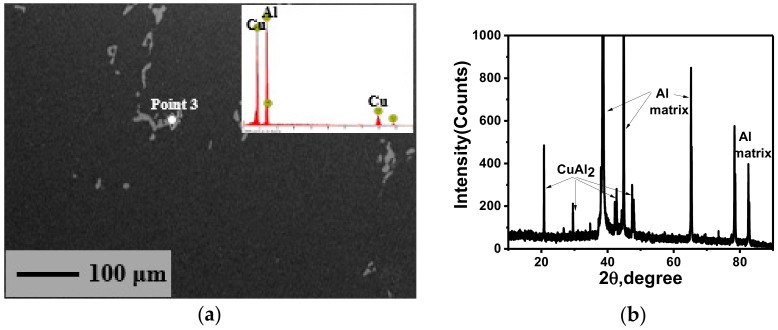
(**a**) The SEM micrograph of the solution-treated samples under the condition of 300 °C-0.01 s^−1^-0.9 with EDS analysis of Point 3; (**b**) XRD analysis of the sample.

The SEM micrograph, EDS analysis, and XRD analysis of the samples after SST are shown in Figure 9. It can be seen that the precipitated phase still exists after SST, and it is determined to be CuAl_2_ phase by EDS and XRD analysis. This is because the content of Cu exceeds the solubility in Al at this temperature. In addition, the previously existing dispersed fine precipitates are all redissolved into the matrix to obtain the supersaturated solid solution for the subsequent aging hardening. However, the second phase remaining is mainly characterized by the form of polymerization, that is to say, large CuAl_2_ lump precipitates cannot be redissolved in the matrix during the SST process, which will have a great impact on the final mechanical performance. Internal cracks can be easily introduced due to the high strength and brittleness of the precipitates polymerization in subsequent processing, thus resulting in defect damage of the component. Therefore, the condensation of CuAl_2_ phase should be avoided and its distribution should be made dispersed in the process of casting and forging for 2219 aluminum alloy, otherwise, the large CuAl_2_ lump precipitates will be inherited in the final products due to the inability of subsequent processing to break down its polymerization state.

## 4. Conclusions

In this study, the approach of “WD + SST” was put forward to investigate the grain refinement of 2219 aluminum alloy, and the thermal compression tests and solid solution treatment experiments were adopted to simulate the process. The effects of deformation parameters on the law of microstructure evolution at various stages during the intermediate thermal-mechanical treatment process were also studied. The following conclusions can be drawn:

1. During the warm deformation process of 2219 aluminum alloy, the flow stress is very sensitive to temperature, strain rate, and strain. The storage energy is found to be proportional to the square of the flow stress, and it decreases with the increase in temperature and/or the decrease in strain rate, and it rises first and then keeps a relatively stable state with the increase in strain. In addition, the main softening mechanism is determined to be dynamic recovery. Under relatively high temperature (270 °C, 300 °C) and lower strain rate (0.01 s^−1^), incomplete continuous dynamic recrystallization can also occur.
2. During the warm deformation process, the grain morphology changes and the substructure content increases. Moreover, the proportion of the low angle grain boundaries increases with the decrease in deforming temperature, the increase in strain rate, and/or the increase in strain. In addition, the distribution of CuAl_2_ phase is more dispersed with the increase in deforming temperature, the decrease in strain rate, and/or the increase in strain. However, some CuAl_2_ phase particles are still polymerized.
3. During the solid solution treatment process of 2219 aluminum alloy, complete static recrystallization occurred and substructure almost disappeared. The average grain size obtained decreased with the decrease in deforming temperature, the increase in strain rate, and/or the increase in strain. The grain refinement mechanism is related to the amount of storage energy and the distribution of precipitated particles in the whole process of intermediate thermal-mechanical treatment. The previously existing dispersed fine precipitates are all redissolved into the matrix, however, the precipitates remaining exist mainly by the form of polymerization.
4. According to the experimental results, the optimum deformation parameters for industrial processing of 2219 aluminum alloy are as follows: T < 240 °C, ε > 0.5, and $\dot{\varepsilon }$ > 1 s^−1^, which can get better grain-refining effects at the same time.

## Figures and Tables

**Table 1:** Chemical composition of the 2219 aluminum alloy studied (mass fraction, %).

Cu	Mn	Si	Zr	Fe	Mg	Zn	V	Ti	Al
5.8–6.8	0.2–0.4	≤0.2	0.1~0.25	≤0.3	≤0.02	0.10	0.05~0.15	0.02~0.1	Bal

**Table 2:** The content of LAGBs and HAGBs under different deformation conditions.

Deformation Parameters	Variables		LAGBs	HAGBs
Initial State	-		77.6%	22.4%
240 °C-0.3 s^−1^	strain	0.2	79.0%	21.0%
		0.5	82.9%	17.10%
		0.9	84.3%	15.7%
0.01 s^−1^-0.9	temperature/°C	210	84.0%	16.0%
		270	77.4%	22.6%
		300	76.8%	23.2%
300 °C-0.9	strain rate/s^−1^	0.01	76.8%	23.2%
		0.1	77.7%	22.3%
		1	84.8%	15.2%

**Table 3:** Grain size deviation under different deformation conditions.

Deformation Parameters	Variables	Average Value of Grain Size, Expectation/μm	Coefficient of Variation(s/EX)	Misorientation Fraction(>15°)
240 °C-0.3 s^−1^	0.2	60	0.72	92.9%
	0.5	35	0.57	94.9%
	0.9	33	0.55	93.4%
0.01 s^−1^-0.9	210 °C	27	0.58	93%
	270 °C	58	0.91	95.5%
	300 °C	87	0.92	93.0%
300 °C-0.9	0.01 s^−1^	87	0.92	93.0%
	0.1 s^−1^	65	0.78	96.3%
	1 s^−1^	32	0.73	93.5%

## References

[1] An L.H., Cai Y., Liu W., Yuan S.J., Zhu S.Q. (2012). Effect of pre-deformation on microstructure and mechanical properties of 2219 aluminum alloy sheet by thermomechanical treatment. Trans. Nonferr. Met. Soc. China.

[2] Venkatesh B.D., Chen D.L., Bhole S.D. (2009). Effect of heat treatment on mechanical properties of Ti-6Al-4V ELI alloy. Mater. Sci. Eng. A.

[3] Zhang J., Chen B., Zhang B. (2012). Effect of initial microstructure on the hot compression deformation behavior of a 2219 aluminum alloy. Mater. Des..

[4] Qu W.Q., Song M.Y., Yao J.S., Zhao H.Y. (2011). Effect of Temperature and Heat Treatment Status on the Ductile Fracture Toughness of 2219 Aluminum Alloy. Mater. Sci. Forum.

[5] Xia C., Zhang W., Kang Z., Jia Y., Wu Y. (2012). High strength and high electrical conductivity Cu-Cr system alloys manufactured by hot rolling–quenching process and thermomechanical treatments. Mater. Sci. Eng. A.

[6] Song R., Ponge D., Raabe D., Speer J.G., Matlock D.K. (2006). Overview of processing, microstructure and mechanical properties of ultrafine grained bcc steels. Mater. Sci. Eng. A.

[7] Liu S., Zhong Q., Zhang Y., Liu W., Zhang X. (2010). Investigation of quench sensitivity of high strength Al-Zn-Mg-Cu alloys by time-temperature-properties diagrams. Mater. Des..

[8] Petch N.J. (1953). The Cleavage Strength of Polycrystals. J. Iron. Steel. Inst..

[9] Moallemi M., Kermanpur A., Najafizadeh A., Rezaee A., Baghbadorani H.S. (2012). Formation of nano/ultrafine grain structure in a 201 stainless steel through the repetitive martensite thermomechanical treatment. Mater. Lett..

[10] Waldman J., Sulinski H., Markus H. (1974). The effect of ingot processing treatments on the grain size and properties of Al alloy 7075. Metall. Trans..

[11] Popov N.N., Prokoshkin S.D., Sidorkin M.Y., Sysoeva T.I., Borovkov D.V. (2007). Effect of thermomechanical treatment on the structure and functional properties of a 45Ti-45Ni-10Nb alloy. Russ. Metall..

[12] Elmay W., Prima F., Gloriant T., Bolle B., Zhong Y. (2013). Effects of thermomechanical process on the microstructure and mechanical properties of a fully martensitic titanium-based biomedical alloy. J. Mech. Behav. Biomed..

[13] Kim H.Y., Ohmatsu Y., Kim J.I., Hosoda H., Miyazaki S. (2006). Mechanical Properties and Shape Memory Behavior of Ti-Mo-Ga Alloys. Mater. Sci. Eng. A.

[14] Russo E.D., Conserva M., Gatto F., Markus H. (1973). Thermo-mechanical treatments on high strength Al-Zn-Mg(-Cu) alloys. Metall. Trans..

[15] Russo E.D., Conserva M., Buratti M., Gatto F. (1974). A new thermo-mechanical procedure for improving the ductility and toughness of Al-Zn-Mg-Cu alloys in the transverse directions. Mater. Sci. Eng..

[16] Lademo O.G., Pedersen K.O., Berstad T., Furu T., Hopperstad O.S. (2008). An experimental and numerical study on the formability of textured AlZnMg alloys. Eur. J. Mech. A-Solid.

[17] Tajally M., Emadoddin E. (2011). Mechanical and anisotropic behaviors of 7075 aluminum alloy sheets. Mater. Des..

[18] Waldman J., Sulinski H.V., Markus H. (1974). Processes for the Fabrication of 7000 Series Aluminum Alloys. U.S. Patent.

[19] Ward B.R., Agrawal S.P., Ashton R.F. (1984). Method of Producing Superplastic Aluminum Sheet. U.S. Patent.

[20] Wert J.A., Paton N.E., Hamilton C.H., Mahoney M.W. (1981). Grain refinement in 7075 aluminum by thermomechanical processing. Metall. Trans. A.

[21] Vandermeer R.A. (2005). Microstructural descriptors and the effects of nuclei clustering on recrystallization path kinetics. Acta. Mater..

[22] Poole W.J., Militzer M., Wells M.A. (2003). Modelling recovery and recrystallization during annealing of AA 5754 aluminum alloy. Met. Sci. J..

[23] Primig S., Leitner H., Knabl W., Lorich A., Clemens H. (2012). Influence of the heating rate on the recrystallization behavior of molybdenum. Mater. Sci. Eng. A.

[24] Yoshida H. (1991). Effect of pre-heat treatment on the superplasticity of a 7475 alloy sheet. J. Jpn. Inst. Light. Met..

[25] Kaibyshev R., Sakai T., Musin F., Nikulin I., Miura H. (2001). Superplastic behavior of a 7055 aluminum alloy. Scripta Mater..

[26] Binlung O.U., Yang J., Yang C. (2007). Effects of Step-Quench and Aging on Mechanical Properties and Resistance to Stress Corrosion Cracking of 7050 Aluminum Alloy. Mater. T. Jim..

[27] Tsai T.C., Chuang T.H. (1997). Role of grain size on the stress corrosion cracking of 7475 aluminum alloys. Mater. Sci. Eng. A.

[28] Zuo J., Hou L., Shi J., Cui H., Zhuang L. (2017). The mechanism of grain refinement and plasticity enhancement by an improved thermomechanical treatment of 7055 Al alloy. Mater. Sci. Eng. A.

[29] Ye L.Y., Zhang X.M., Liu Y.W., Tang J.G., Zheng D.W. (2014). Effect of two-step aging on recrystallized grain size of Al-Mg-Li alloy. Mater. Sci. Technol..

[30] Troeger L.P., Jr E.A.S. (2000). Microstructural and mechanical characterization of a super-plastic 6xxx aluminum alloy. Mater. Sci. Eng. A.

[31] Esmaeili S., Lloyd D.J., Jin H. (2011). A thermomechanical process for grain refinement in precipitation hardening AA6xxx aluminum alloys. Mater. Lett..

[32] Aboutalebi M.R., Karimzadeh M., Salehi M.T., Abbaasi S.M., Morakabati M. (2015). Influences of Aging and Thermomechanical Treatments on the Martensitic Transformation and Superelasticity of Highly Ni-rich Ti-51.5 at.% Ni Shape Memory Alloy. Thermochim. Acta.

[33] Ozan S., Lin J., Li Y., Zhang Y., Munir K. (2018). Deformation mechanism and mechanical properties of a thermomechanically processed β Ti-28Nb-35.4Zr alloy. J. Mech. Behav. Biomed..

[34] Saastamoinen A., Kaijalainen A., Porter D., Suikkanen P. (2017). The effect of thermomechanical treatment and tempering on the subsurface microstructure and bendability of direct-quenched low-carbon strip steel. Mater. Charact..

[35] Misra R.D.K., Kumar B.R., Somani M., Karjalainen P. (2008). Deformation processes during tensile straining of ultrafine/nanograined structures formed by reversion in metastable austenitic steels. Scripta Mater..

[36] Eskandari M., Kermanpur A., Najafizadeh A. (2009). Formation of Nanocrystalline Structure in 301 Stainless Steel Produced by Martensite Treatment. Metall. Mater. Trans. A.

[37] Forouzan F., Najafizadeh A., Kermanpur A. (2010). Production of nano/submicron grained AISI 304L stainless steel through the martensite reversion process. Mater. Sci. Eng. A.

[38] Chen F., Cui Z., Chen S. (2011). Recrystallization of 30Cr2Ni4MoV Ultra-super-critical Rotor Steel during Dot Deformation Part 3 Metadynamic Recrystallization. Mater. Sci. Eng. A.

[39] Lin Y.C., Chen M.S., Zhong J. (2008). Microstructural evolution in 42CrMo steel during compression at elevated temperatures. Mater. Lett..

[40] Momeni A., Dehghani K. (2010). Prediction of dynamic recrystallization kinetics and grain size for 410 martensitic stainless steel during hot deformation. Met. Mater. Int..

[41] Lin Y.C., Chen X.M., Wen D.X., Chen M.S. (2014). A physically-based constitutive model for a typical nickel-based superalloy. Comp. Mater. Sci..

[42] Khelfaoui F., Guénin G. (2003). Influence of the recovery and recrystallization processes on the martensitic transformation of cold worked equiatomic Ti–Ni alloy. Mater. Sci. Eng. A.

[43] Honeycombe R.W.K., Bhadeshia H. (1981). Pobert William Kerr. Steels: Microstructure and Properties.

[44] Peng X.N., Guo H.Z., Shi Z.F., Qin C., Zhao Z.L. (2014). Microstructure characterization and mechanical properties of TC4-DT titanium alloy after thermomechanical treatment. T. Nonferr. Metal. Soc..

[45] Wert J.A., Austin L.K. (1988). Modeling of thermo-mechanical processing of heat-treatable aluminum alloys. Metall. Trans. A.

[46] Nie W., Shang C. (2012). Microstructure and toughness of the simulated welding heat affected zone in XIOO pipeline steel with high deformation resistance. Acta. Metall. Sin..

[47] Sha G., Wang Y.B., Liao X.Z., Duan Z.C., Ringer S.P. (2009). Influence of equal-channel angular pressing on precipitation in an Al-Zn-Mg-Cu alloy. Acta. Mater..

[48] Deschamps A., Fribourg G., Brechet Y., Chemin J.L., Hutchinson C.R. (2012). In situ evaluation of dynamic precipitation during plastic straining of an Al-Zn-Mg-Cu alloy. Acta. Mater..

[49] Lang Y., Zhou G., Hou L., Zhang J., Zhuang L. (2015). Significantly enhanced the ductility of the fine-grained Al-Zn-Mg-Cu alloy by strain-induced precipitation. Mater. Des..

[50] Kita K., Kobayashi K., Monzen R. (2000). Dispersions of Particles and Heat Resistance in a Cu-Fe-P Alloy (Special Issue on Composite Materials). J. Soc. Mater. Sci..

[51] Huo W., Hou L., Lang Y., Cui H., Zhuang L. (2015). Improved thermo-mechanical processing for effective grain refinement of high-strength AA 7050 Al alloy. Mater. Sci. Eng. A.

[52] Zuo J., Hou L., Shi J., Cui H., Zhuang L. (2016). Precipitates and the evolution of grain structures during double-step rolling of high-strength aluminum alloy and related properties. Acta. Metall. Sin..

[53] Yang H.S., Mukherjee A.K., Roberts W.T. (1992). Effect of cooling rate in over-ageing and other thermomechanical process parameters on grain refinement in an AA7475 aluminum alloy. J. Mater. Sci..

[54] Hall E.O. (1951). The Deformation and Ageing of Mild Steel: II Characteristics of the Lüders Deformation. Proc. Phys. Soc..

[55] Wert J.A. (1980). Strength of Metals and Alloys.

[56] Ma K., Wen H., Hu T., Topping T.D., Isheim D. (2014). Mechanical behavior and strengthening mechanisms in ultrafine grain precipitation-strengthened aluminum alloy. Acta. Mater..

[57] Yang Y., Yu K., Li Y., Zhao D., Liu X. (2012). Evolution of nickel-rich phases in Al-Si-Cu-Ni-Mg piston alloys with different Cu additions. Mater. Des..

[58] Gourdet S., Montheillet F. (2000). An experimental study of the recrystallization mechanism during hot deformation of aluminum. Mater. Sci. Eng. A.

[59] Sitdikov O., Sakai T., Miura H., Hama C. (2009). Temperature effect on fine-grained structure formation in high-strength Al alloy 7475 during hot severe deformation. Mater. Sci. Eng. A.

